# Use of a taxon-specific reference database for accurate metagenomics-based pathogen detection of *Listeria monocytogenes* in turkey deli meat and spinach

**DOI:** 10.1186/s12864-023-09338-w

**Published:** 2023-06-27

**Authors:** Jillian Rumore, Matthew Walker, Franco Pagotto, Jessica D. Forbes, Christy-Lynn Peterson, Andrea D. Tyler, Morag Graham, Gary Van Domselaar, Celine Nadon, Aleisha Reimer, Natalie Knox

**Affiliations:** 1 Department of Medical Microbiology and Infectious Diseases, University of Manitoba, Winnipeg, MB Canada; 2 Public Health Agency of Canada, National Microbiology Laboratory, MB Winnipeg, Canada; 3 Food Directorate, Health Canada, Bureau of Microbial Hazards, Ottawa, ON Canada; 4 Eastern Ontario Regional Laboratory Association, Ottawa, ON Canada

**Keywords:** Reference database, Pathogen detection, *Listeria monocytogenes*, Metagenomics classification, Foodborne outbreak

## Abstract

**Background:**

The reliability of culture-independent pathogen detection in foods using metagenomics is contingent on the quality and composition of the reference database. The inclusion of microbial sequences from a diverse representation of taxonomies in universal reference databases is recommended to maximize classification precision for pathogen detection. However, these sizable databases have high memory requirements that may be out of reach for some users. In this study, we aimed to assess the performance of a foodborne pathogen (FBP)-specific reference database (taxon-specific) relative to a universal reference database (taxon-agnostic). We tested our FBP-specific reference database's performance for detecting *Listeria monocytogenes* in two complex food matrices—ready-to-eat (RTE) turkey deli meat and prepackaged spinach—using three popular read-based DNA-to-DNA metagenomic classifiers: Centrifuge, Kraken 2 and KrakenUniq.

**Results:**

In silico host sequence removal led to substantially fewer false positive (FP) classifications and higher classification precision in RTE turkey deli meat datasets using the FBP-specific reference database. No considerable improvement in classification precision was observed following host filtering for prepackaged spinach datasets and was likely a consequence of a higher microbe-to-host sequence ratio. All datasets classified with Centrifuge using the FBP-specific reference database had the lowest classification precision compared to Kraken 2 or KrakenUniq. When a confidence-scoring threshold was applied, a nearly equivalent precision to the universal reference database was achieved for Kraken 2 and KrakenUniq. Recall was high for both reference databases across all datasets and classifiers. Substantially fewer computational resources were required for metagenomics-based detection of *L. monocytogenes* using the FBP-specific reference database, especially when combined with Kraken 2.

**Conclusions:**

A universal (taxon-agnostic) reference database is not essential for accurate and reliable metagenomics-based pathogen detection of *L. monocytogenes* in complex food matrices. Equivalent classification performance can be achieved using a taxon-specific reference database when the appropriate quality control measures, classification software, and analysis parameters are applied. This approach is less computationally demanding and more attainable for the broader scientific and food safety communities.

**Supplementary Information:**

The online version contains supplementary material available at 10.1186/s12864-023-09338-w.

## Background

Detecting pathogenic microorganisms in foods requires fast and reliable techniques to identify contamination sources to mitigate foodborne outbreaks and prevent the further spread of illness [1]. Complex food matrices contain a heterogeneous mixture of resident microbiota, inorganic particles, and biochemical components. These matrices pose additional challenges for detecting pathogens, which can be present at low levels but in sufficient numbers to cause illness. Culture-enrichment techniques are often required to increase target pathogen levels to a degree sufficient for detection and isolation, but these techniques are laborious and can introduce delays depending on the growth characteristics of the target pathogen [1, 2]. Metagenomics—the direct sequencing of all DNA present in a food sample without pathogen-specific isolation—has already proven useful for many applications along the foodborne disease continuum, including taxonomic profiling of complex microbial populations in various food matrices [3–5], informing culture-based enrichment strategies via comprehensive characterization of background microbiota population dynamics [6, 7], detection of non-culturable, fastidious and/or potential emerging pathogens [8–10], and detecting enteric foodborne pathogens in clinical specimens [11, 12] and foods [13–16].

This rapidly evolving laboratory tool has the potential to modernize food safety management and increase the speed and scope for the detection of contamination and outbreak investigations. However, metagenomics is currently not an approved method for microbiological detection in the food processing environment in North America. Many challenges exist and must be overcome before this approach can be routinely implemented by regulatory agencies for detection and characterization of pathogens in food. While most of these challenges are inherent to the wet-laboratory protocol and have been discussed elsewhere [2, 17], careful consideration must also be given to the data analysis component, which is critically dependent on the reference database. This is because metagenomics-based pathogen detection is primary accomplished using classification software to assign taxonomic identity to the reads (or assembled contigs) in the sequence dataset by matching them against previously sequenced microbial genomes contained in reference databases [18]. As such, the reliability of metagenomics-based pathogen detection is contingent on the quality and composition of the reference database [19].

Low-complexity sequences, contamination in published genomes from human and non-human sources, and lack of diversity in the reference databases have been reported to contribute to false-positive (FP) classifications in metagenomics-based studies [19–23]. In the context of food safety, a FP result occurs when a pathogen is absent in the food but is "detected" by the analysis method. This scenario can result in a recall of a pathogen-free product and, subsequently, lost revenue and food waste. False negatives (FN) can also arise (i.e., a pathogen is present in the food, but the test result shows it is not detected) and are often a consequence of insufficient taxonomic diversity in reference databases (i.e., lack of an appropriate reference genome). This issue has been exacerbated by sequencing efforts primarily targeting common human pathogens, leaving rare pathogens underrepresented in genomic reference databases [19, 24]. The inability to detect the pathogen increases the risk of foodborne illness or outbreaks as contaminated products would potentially remain on the market. However, FN classifications are extremely challenging to decipher in real-world metagenomic datasets as the negative class typically contains "unknown unknowns" [25]. Hence, the focus for metagenomic classification has been primarily centered on characterizing the positive class of identified taxa as it can be more easily quantified [25].

To minimize the likelihood of FP classifications, the use of reference databases containing all domains of life (i.e., taxon-agnostic) has been recommended, even if the focus is on a particular taxonomic group [26]. However, this is not feasible for at least two reasons: 1) genomes contained in sequence repositories do not accurately reflect the composition of the natural world, and 2) high memory requirements for large reference databases (10-100 s of gigabytes) would be prohibitive in settings without access to a high-performance computing environment [24, 25, 27]. To address these limitations, smaller, taxon-specific reference databases may be necessary. However, the suitability of such databases for metagenomic-based foodborne pathogen detection has not been assessed. Such an evaluation is especially important for high-consequence pathogens like *Listeria monocytogenes*, whose outbreaks are costly and often lead to high case-fatality rates (~ 20–30%), particularly among persons with weakened immune systems [28, 29].

Due to the severe public health consequences of listeriosis, the occurrence of *L. monocytogenes* in the food processing environment (FPE), which can persist for years or even decades, has the potential for broad and rapid spread through the food system and is of primary concern [30–32]. Persistence in the FPE is especially problematic for the ready-to-eat (RTE) food industry as these foods do not require further preparation between production and consumption, with the exception of washing/rinising, thawing or warming [32]. For example, one of the first *L. monocytogenes* illness outbreaks attributed to contamination originating from the processing environment was linked to a RTE meat processing facility in 1998–1999, which resulted in 108 illnesses, 14 adult deaths and four miscarriages [33]. Since then, the FPE has been implicated in other listeriosis outbreaks including a multiprovince outbreak that occurred in Canada in 2008, which led to 57 illnesses and 24 deaths due to RTE delicatessen (deli) meat [34]. Despite the historical association of *L. monocytogenes* outbreaks with RTE meats, more recent outbreaks of listeriosis have been linked to the consumption of fresh produce, including a multistate crossborder outbreak of listeriosis associated with packaged leafy greens that occurred in 2015–2016 in the United States and Canada [35]. Unlike RTE meat, fresh produce is minimally processed (i.e., washed, santizied, packaged) and is not subjected to additional processing steps that would further reduce the microbiological burden and eliminate harmful pathogens [36]. Therefore, timely and accurate detection is of utmost importance.

In this pilot study, we used precision and recall—two widely used metrics for metagenomic classification—to assess the performance of a foodborne pathogen (FBP)-specific reference database (taxon-specific) compared to a universal reference database (taxon-agnostic) for detecting *L. monocytogenes* in artificially contaminated foods: ready-to-eat (RTE) turkey deli meat and prepackaged fresh spinach. We compared three popular read-based DNA-to-DNA classifiers: Centrifuge, Kraken 2 and KrakenUniq [37–39]. These tools assign taxonomic labels to reads by exact matching of short nucleotide segments of a predefined length (*k-*mer) against a database consisting of reference genomes and their corresponding taxonomic identifications [18]. Our goal was to determine if equivalent precision, defined herein as the proportion of classifications that are true positives over the number of positive calls, and recall (defined as the proportion of classifications that are true positives over the ground truth) could be achieved for the FBP-specific reference database when the appropriate quality control measures, classification tools and analysis parameters are applied. To minimize classification performance bias attributed to differences in reference database composition, we custom-built a FBP-specific reference database and a universal reference database with consistent reference sequences and taxonomy across all classifiers [25].

## Results

### Provision of test datasets

An average of 1,025,280 paired-end (PE) reads were generated for RTE turkey deli meat datasets using the Qiagen QIAamp® Fast DNA Stool Mini Kit (Qiagen, Valencia, California), which was 8–11% higher than the average number of PE reads generated for datasets processed with the Qiagen DNeasy® PowerSoil Kit (Qiagen, Valencia, California) (average 948,890 PE reads) or the Zymo Research Quick-DNA Fecal/Soil Microbe Miniprep Kit (Zymo Research, Irvine, CA) (average 923,429 PE reads). As for prepackaged spinach datasets, 6% more PE reads were generated when using the Qiagen DNeasy® PowerSoil Kit (average 964,671) compared to Qiagen QIAamp® Fast DNA Stool Mini Kit (average 902,795 PE reads), and 75% more compared to the Zymo Research Quick-DNA Fecal/Soil Microbe Miniprep Kit (average 551,484 PE reads). Although the lowest number of PE reads was generated for datasets extracted with the Zymo Research Quick-DNA Fecal/Soil Microbe Miniprep Kit across both foods types, the average proportion of reads classified as *L. monocytogenes* was highest for these datasets (Additional file 1). This finding was likely attributed to the more efficient lysis of Gram-positive bacteria compared to the other commercial kits. Since an in-depth evaluation of the DNA extraction kits for the detection of *L. monocytogenes* in foods was not an objective of this pilot study, we conducted our evaluation using datasets extracted with the Zymo Research Quick-DNA Fecal/Soil Microbe Miniprep Kit to simplify the analysis and eliminate a potential confounder associated with differences in DNA extraction efficiency.

### Including an in silico host DNA removal step can minimize false-positive classifications when using a taxon-specific reference database

**Fig. 1:**
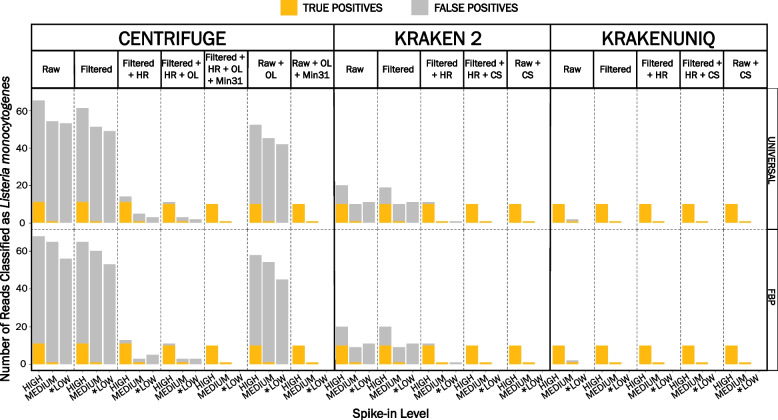
Comparison of the average number of reads classified as *Listeria monocytogenes* in artificially contaminated ready-to-eat (RTE) turkey deli meat. RTE turkey deli meat was artificially contaminated with *L. monocytogenes* colony forming units (CFU) at three levels (high:10^4^ CFU/ml; medium:10^3^ CFU/ml; low:10^2^ CFU/ml). Genomic DNA was extracted in duplicate with the Zymo Research Quick-DNA Fecal/Soil Microbe Miniprep Kit. The number of reads classified as *L. monocytogenes* was averaged across reference databases and classifiers for each set of replicates. Raw = raw datasets classified with default settings (no host sequence removal); Filtered = low-quality read filtered datasets (fastp) classified with default settings; Filtered + HR = low-quality read filtered (fastp) and in silico host removed (Bowtie 2) datasets classified with default settings; Filtered + HR + OL = low-quality read filtered (fastp) and in silico host removed (Bowtie 2) datasets classified using Centrifuge with the sequence threshold label adjusted to one (*k* = 1) to mimic the classification algorithm of Kraken 2 and KrakenUniq; Filtered + HR + OL + Min31 = low-quality read filtered (fastp) and in silico host removed (Bowtie 2) datasets classified using Centrifuge with the sequence threshold label adjusted to one (*k* = 1) to mimic the classification algorithm of Kraken 2 and KrakenUniq and minimum length of partial hits adjusted to 31 (*–min-hitlen* = 31); Raw + OL = raw datasets classified with one taxonomic label (*k* = 1); Raw + OL + Min31 = raw datasets classified using Centrifuge with the sequence threshold label adjusted to one (*k* = 1) to mimic the classification algorithm of Kraken 2 and KrakenUniq and minimum length of partial hits adjusted to 31 (*–min-hitlen* = 31); Filtered + HR + CS = low-quality read filtered (fastp) and in silico host removed (Bowtie 2) datasets classified with Kraken 2 or KrakenUniq and a confidence score threshold of 0.1 (*–confidence* = 0.1); Raw + CS = raw datasets classified with Kraken 2 or KrakenUniq and a confidence score threshold of 0.1 (*–confidence* = 0.1). UNIVERSAL = universal (taxon-agnostic) reference database; FBP = foodborne pathogen-specific (taxon-specific) reference database. *No TP reads were detected

Compared to the *L. monocytogenes* genome (~ 3.0 million bp), the turkey genome (*Melagris gallopavo*) is around 1000 times larger (~ 1.1 billion bp) [40], while the spinach genome (*Spinacea oleracea*) is around 100 times larger (~ 990 million bp) [41]. Therefore, the primary food matrix can introduce a large quantity of "host-derived" DNA that can obscure the detection of low-level pathogens. As expected, the proportion of FP reads in RTE turkey deli meat raw datasets varied across metagenomic classifiers but was generally higher for low spike-in raw datasets than the medium and high spike-in raw datasets (Fig. 1, Additional file 2). Across both reference databases, 56 FP reads were detected in RTE turkey deli meat raw datasets classified with Centrifuge compared to ≤ 11 FP reads when classified with Kraken 2 and ≤ 1 FP read when classified with KrakenUniq (Fig. 1, Additional file 2). Including a data quality filtering step to remove adaptor contamination, low-quality bases, and short reads using fastp did not substantially impact the number of FP classifications for either reference database. However, the preprocessing of raw datasets is a prerequisite step to improve data quality for downstream analysis, and is highly recommended. On average, FP reads were reduced by < 8% (≤ 5 reads) in RTE turkey deli meat datasets classified with either Centrifuge or Kraken 2 for both the FBP-specific and universal reference databases (Fig. 1, Additional file 2). A single FP classification was resolved in the medium spike-in RTE turkey deli meat dataset when KrakenUniq was used. Including an in silico host sequence removal step to low-quality read filtered datasets reduced FP reads by 90% or more (~ 50 reads) in RTE turkey deli meat datasets across both reference databases when classified with either Centrifuge or Kraken 2 (Fig. 1, Additional file 2). The FP reads were investigated using the Basic Local Alignment Search Tool (BLAST) and the National Center for Biotechnology Information (NCBI) nucleotide (nt) database [42, 43]. BLAST analysis revealed that FP reads predominately aligned to the turkey genome (*M. gallopavo;* GCF_000146605.3) or a close descendant thereof; this was not unexpected since, on average, > 96% of the total reads were host-derived (Additional file 3).

Reference database contamination due to sequence mislabeling can lead to misclassifications in metagenomics analyses. Therefore, to rule out cross-kingdom contamination between the turkey genome and the *L. monocytogenes* genomes included in the reference databases, conterminator was used to perform an all-against-all sequence comparison [44]. The conterminator analysis did not identify contamination between the two taxa (Additional file 4). This finding was not surprising given recent efforts to remove contaminated sequences from the turkey genome, which was once considered the most contaminated genome in the Reference Sequence Database (RefSeq) [44].

**Fig. 2:**
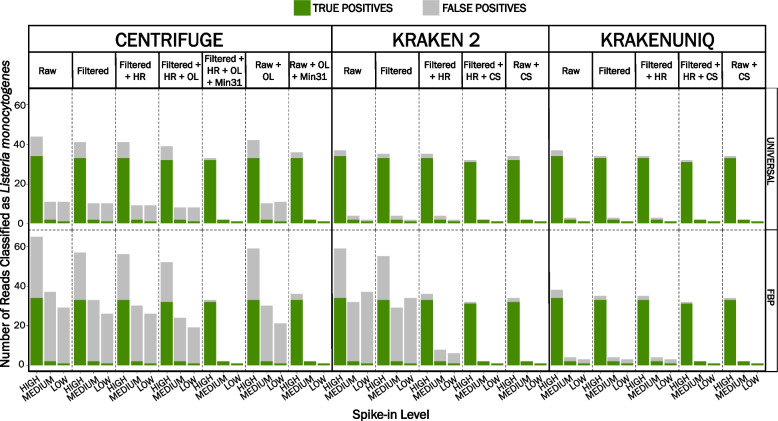
Comparison of the average number of reads classified as *Listeria monocytogenes* in artificially contaminated prepackaged spinach. Prepackaged spinach was artificially contaminated with *L. monocytogenes* colony forming units (CFU) at three levels (high:10^4^ CFU/ml; medium:10^3^ CFU/ml; low:10^2^ CFU/ml). Genomic DNA was extracted in duplicate with the Zymo Research Quick-DNA Fecal/Soil Microbe Miniprep Kit. The number of reads classified as *L. monocytogenes* was averaged across reference databases and classifiers for each set of replicates. Raw = raw datasets classified with default settings (no host sequence removal); Filtered = low-quality read filtered datasets (fastp) classified with default settings; Filtered + HR = low-quality read filtered (fastp) and in silico host removed (Bowtie 2) datasets classified with default settings; Filtered + HR + OL = low-quality read filtered (fastp) and in silico host removed (Bowtie 2) datasets classified using Centrifuge with the sequence threshold label adjusted to one (*k* = 1) to mimic the classification algorithm of Kraken 2 and KrakenUniq; Filtered + HR + OL + Min31 = low-quality read filtered (fastp) and in silico host removed (Bowtie 2) datasets classified using Centrifuge with the sequence threshold label adjusted to one (*k* = 1) to mimic the classification algorithm of Kraken 2 and KrakenUniq and minimum length of partial hits adjusted to 31 (*–min-hitlen* = 31); Raw + OL = raw datasets classified with one taxonomic label (*k* = 1); Raw + OL + Min31 = raw datasets classified using Centrifuge with the sequence threshold label adjusted to one (*k* = 1) to mimic the classification algorithm of Kraken 2 and KrakenUniq and minimum length of partial hits adjusted to 31 (*–min-hitlen* = 31); Filtered + HR + CS = low-quality read filtered (fastp) and in silico host removed (Bowtie 2) datasets classified with Kraken 2 or KrakenUniq and a confidence score threshold of 0.1 (*–confidence* = 0.1); Raw + CS = raw datasets classified with Kraken 2 or KrakenUniq and a confidence score threshold of 0.1 (*–confidence* = 0.1). UNIVERSAL = universal (taxon-agnostic) reference database; FBP = foodborne pathogen-specific (taxon-specific) reference database

Similar to the RTE turkey deli meat datasets, the proportion of FP classifications in prepackaged spinach datasets varied across metagenomic classifiers and was consistently higher for low spike-in datasets (Fig. 2, Additional file 2). A higher proportion of FP classifications was observed in datasets classified with the FBP-specific reference database compared to the universal reference database, particularly when using Centrifuge or Kraken 2. On average, 30 FP reads were identified in prepackaged spinach datasets when classified with Centrifuge or Kraken 2 using the FBP-specific reference database compared to ≤ 10 FP reads for datasets classified with Centrifuge and ≤ 3 FP reads for datasets classified with Kraken 2 using the universal reference database (Fig. 2, Additional file 2). Regardless of the reference database used, fewer than five FP reads were detected in unfiltered prepackaged spinach datasets when classified with KrakenUniq.

Filtering low-quality reads with fastp led to a maximum 21% reduction (maximum 7 reads) in FP reads for prepackaged spinach datasets classified with Centrifuge and the FBP-specific reference database compared to a 16% reduction (maximum 2 reads) when using the universal reference database (Fig. 2, Additional file 2). Across the same datasets, a 10% reduction in FP reads (3 reads) was observed when using Kraken 2 and the FBP-specific reference database. No change in FP reads occurred in the medium and low spike-in datasets classified with Kraken 2 and the universal database, whereas a 33% decrease in FP reads occurred in the high spike-in dataset (1 read). When classified with KrakenUniq, FP reads were reduced by 59% (2 reads) in the high spike-in dataset across both reference databases (Fig. 2, Additional file 2). A more substantial decrease in FP reads was observed when the taxonomic classification was performed on preprocessed (i.e., low-quality read filtered and in silico removal of host DNA) prepackaged spinach datasets, particularly when using Kraken 2 and the FBP-specific reference database. In these datasets, FP reads were reduced by an average of 85% (23 reads) compared to 21% (2 reads) when classified with Centrifuge across both reference databases. The number of TP reads did not change for datasets classified with Kraken 2 or KrakenUniq and was consistent across reference databases (Fig. 2, Additional file 2).

Unlike the RTE turkey deli meat datasets, which consisted almost entirely of host DNA (> 96%), less than 7% of the total sequenced PE reads aligned to the spinach genome (Additional file 3). Therefore, it was unsurprising that including a host-removal step generally had a lower impact on the number of FP reads for these datasets. BLAST analysis against the nt database revealed that FP reads detected in prepackaged spinach datasets classified with the universal reference database primarily aligned to the lettuce genome (*Lactuca sativa*), which we suspect was better represented in the NCBI nt database compared to the spinach genome (*Spinacia oleracea)*. Though several FP reads detected in prepackaged spinach datasets classified with the FBP-specific reference database also aligned to the lettuce genome, the majority of FP reads aligned to the soil-ubiquitous genera *Pseudomonas*, which is known to colonize spinach [5, 45]. When using the universal reference database, ≥ 50% of the total reads were classified as *Pseudomonas* spp*.* in the prepackaged spinach datasets across all classifiers. Since these genera were not included in the FBP-specific reference database, true *Pseudomonas* spp. reads were associated with an identical or similar region also present in a distantly related taxon in the database (i.e., *L. monocytogenes*), leading to misclassification [26].

Using conterminator, cross-kingdom contamination was predicted between the *L. monocytogenes* reference genomes and the lettuce genome (GCF_002870075.2), specifically, the chloroplast genome (NC_007578.1) and ribosomal RNA (rRNA) regions in *L. monocytogenes* (Additional file 4). Due to the high similarity between plastid and bacterial rRNAs, erroneous integration of bacterial sequences into chloroplast ribosomal RNA is not uncommon [46].

**Fig. 3:**
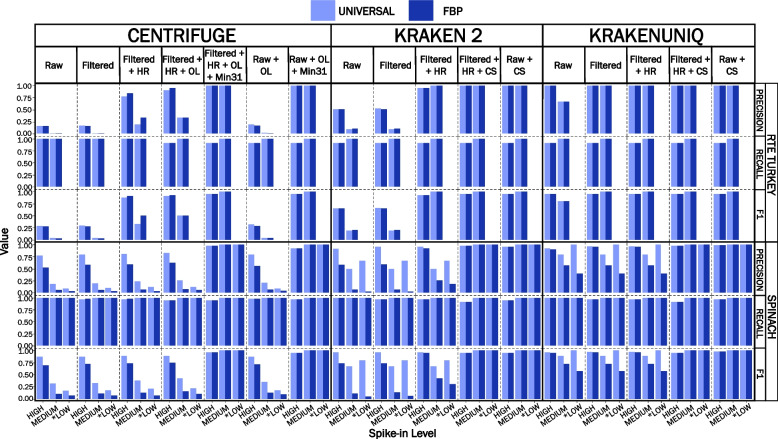
Comparison of precision, recall and F1 score across reference databases and classifiers. Genomic DNA was extracted in duplicate with the Zymo Research Quick-DNA Fecal/Soil Microbe Miniprep Kit. The number of reads classified as *Listeria monocytogenes* was averaged across reference databases and classifiers for each set of replicates. Precision, recall and F1-score were calculated for samples artificially contaminated with *L. monocytogenes* colony forming units (CFU) at three levels (high:10^4^ CFU/ml; medium 10³ CFU/ml; low: 10² CFU/ml). Raw = raw datasets classified with default settings (no host sequence removal); Filtered = low-quality read filtered datasets (fastp) classified with default settings; Filtered + HR = low-quality read filtered (fastp) and in silico host removed (Bowtie 2) datasets classified with default settings; UNIVERSAL = universal (taxon-agnostic) reference database; FBP = foodborne pathogen-specific (taxon-specific) reference database. *No *L. monocytogenes* reads were detected in RTE turkey deli meat low spike-in datasets

Preprocessing the data improved classification precision across both reference databases (Fig. 3, Additional file 2). For RTE turkey deli meat datasets, the precision increased from < 0.52 in raw datasets to > 0.95 for preprocessed datasets when using Kraken 2, and was equivalent across reference databases. Data preprocessing did not affect the precision for high and low spike-in RTE turkey deli meat datasets classified with KrakenUniq as it was already optimal (1.0), but it did improve the precision from 0.67 to 1.0 for the medium spike-in RTE turkey deli meat dataset (Fig. 3, Additional file 2). This finding was consistent across reference databases. Although a noticeable improvement in precision was observed, preprocessed RTE turkey deli meat datasets classified with Centrifuge still had the lowest precision (< 0.85) across both reference databases. When using the universal reference database, the precision increased from 0.92 to 0.96 for high spike-in prepackaged spinach datasets classified with Kraken 2 and from 0.93 to 0.97 when classified with KrakenUniq. No change in precision was observed in medium and low spike-in datasets across the same classifiers and universal reference database (Fig. 3, Additional file 2).

When using the FBP-specific reference database, a more substantial increase in precision from 0.58 to 0.93 was observed for preprocessed prepackaged spinach datasets classified with Kraken 2 compared to a minor increase in precision from 0.91 to 0.96 when using KrakenUniq. No change in precision was observed across the same datasets when classified with Centrifuge, regardless of the reference database. Recall remained high across reference databases for all classifiers (> 0.90), and the F1 score was highest for datasets classified with the universal reference database (Fig. 3, Additional file 2).

### Metagenomic classifier and analysis parameters influence the number of false-positive classifications when using a taxon-specific reference database

Unlike Kraken 2 and KrakenUniq, which assign a single taxonomic label per read using the lowest common ancestor approach (LCA), Centrifuge, by default, assigns up to five taxonomic labels per read using the Burrows-Wheeler transform (BWT) and Ferragina-Mazini (FM) index [37]. To emulate the behavior of Kraken 2 and KrakenUniq, Centrifuge's sequence threshold label (*k*) was adjusted from five (*k *= 5) to one (*k* = 1) to assign a single taxonomic label per read using the LCA [37]. After applying this parameter to low-quality read filtered and in silico host-removed datasets, a minor improvement in Centrifuge's FP reads was observed. For high spike-in RTE turkey deli meat datasets classified with Centrifuge and *k* = 1, FP reads decreased by 33–100% (≤ 3 reads) across both reference databases and the precision increased to > 0.75 (Fig. 3, Additional file 2). Unfortunately, the precision for medium and low spike-in RTE turkey deli meat datasets remained low (≤ 0.33). For prepackaged spinach datasets classified with Centrifuge (*k* = 1) and the universal reference database, FP reads decreased by 15% or less (1 read) compared to ≤ 27% (≤ 7 reads) when using the FBP-specific reference database (Fig. 2, Additional file 2). As a result, the precision did not noticeably improve, and was low (< 0.65) across both reference databases for nearly all prepackaged spinach datasets except for the high spike-in dataset, where the precision was approximately 0.83 (Fig. 3, Additional file 2). The recall decreased slightly in high-spike in datasets for both RTE turkey deli meat (0.91) and prepackaged spinach (0.94) datasets classified with Centrifuge and the aforementioned parameters but remained unchanged in medium and low spike-in datasets across both sample types (Fig. 3, Additional file 2).

In contrast to Kraken 2 and KrakenUniq, which use a single default *k-*mer length of 35 bp and 31 bp, respectively, Centrifuge makes use of both large (i.e., ≥ 31 bp) and small (i.e., 20–25 bp) *k-*mers to achieve a more desirable trade-off between sensitivity and precision [37]. Since Centrifuge can assign taxonomic identity based on exact *k*-mer matches of at least 22 bp, we suspected the low precision was attributed to these smaller *k*-mer matches [37]. To confirm our suspicions, we adjusted the minimum length of partial hits *(--min-hitlen*) parameter from a default of 22 bp to 31 bp and re-analyzed the data with *k* = 1 to assign a single taxonomic label per read using the LCA approach. Using the modified Centrifuge parameters, the precision (≥ 0.98) and recall (> 0.90) were found to be equivalent across reference databases and nearly identical to Kraken 2 and KrakenUniq (Fig. 3, Additional file 2). Even when applying the same modified Centrifuge parameters to raw datasets, high precision (> 0.92) and recall (> 0.90) were still achieved for both reference databases (Fig. 3, Additional file 2).

Different from KrakenUniq, where all *k-*mer information is indexed in a large reference database, Kraken 2 only indexes a small fraction of this information in the form of minimizers, which is a representative sequence of a group of highly similar *k*-mers [30]. Since less information is stored in Kraken-2-indexed databases, more FP classifications (although minimal) are possible and were evident in this study. To determine whether classification precision could be further improved for the FBP-specific reference database, we adjusted the confidence score threshold (--*confidence*) from a default of zero to 0.1. This means that at least 10% of the read's *k-*mer evidence must support the lowest taxonomic rank assigned; otherwise, the read is assigned to a higher taxonomic rank meeting the specified *k*-mer threshold [47]. In this study, a threshold of 0.1 was sufficient to resolve nearly all FP classifications using Kraken 2 or KrakenUniq and increased classification precision (≥ 0.98) across both reference databases (Fig. 3, Additional file 2). A single FP read remained in the high spike-in prepackaged spinach dataset but was resolved by increasing the confidence score threshold to 0.2. When a confidence score threshold of 0.1 was applied to raw datasets, a high level of precision was also attainted (> 0.98), albeit slightly lower for datasets classified with Kraken 2 (≥ 0.95) (Fig. 3, Additional file 2).

While adjusting the analysis parameters improved classification precision across reference databases and classifiers, a slight loss in sensitivity and recall was observed. On average, ≤ 6% of TP classifications (≤ 2 reads) were lost to read reclassifications to a higher taxonomic rank (e.g., *Listeria* species or higher) as a result of insufficient *k-*mer evidence (confidence threshold of 0.1) to support a taxonomic label of *L. monocytogenes*. The F1 score remained high (≥ 0.95) and was equivalent across reference databases for each classifier (Fig. 3, Additional file 2).

### Detection of L. monocytogenes in RTE turkey deli meat and prepackaged spinach is challenging without culture enrichment

Overall, the number of TP reads was equivalent across reference databases for each classifier (Figs. 1 and 2 and Additional file 2). An average of ten TP reads were detected in high spike-in RTE turkey deli meat datasets compared to a single read in medium spike-in and no reads in low spike-in datasets. Though slightly higher, an average of 31 TP reads were detected in high spike-in prepackaged spinach datasets compared to two reads and one read in medium and low spike-in datasets, respectively. These findings highlight the need for culture enrichment, the first step in pathogen detection and recovery from foods, to increase the target pathogen to levels sufficient for food safety monitoring and outbreak response activities, especially in foods with low contamination levels. Interestingly, recent evidence suggests that using read counts for pathogen detection can be misleading, particularly for detecting low pathogen levels [39].

To address this concern, the developers of Kraken 2 recently incorporated distinct-counting estimation of minimizers, a feature leveraged from KrakenUniq (described as unique *k*-mers) that aims to improve pathogen detection accuracy [48]. In essence, distinct *k-*minimizers are used as a proxy for genome coverage. For example, taxonomic classifications with a higher number of distinct *k*-minimizers indicate the reads are more evenly distributed across the genome, suggesting the pathogen is likely present. In contrast, a low number of distinct *k-*minimizers suggests the reads are concentrated in a single or very few locations in the genome and likely a false-positive identification [39]. Since accurate detection of *L. monocytogenes* represents an enormous public benefit and < 0.1% of the total sequenced reads were classified as *L. mono*cytogenes in our study datasets, we wanted to explore the usefulness of distinct *k*-minimizers and how they compared between reference databases for preprocessed datasets. We specifically focused on distinct *k-*minimizers reported by Kraken 2 as the number of unique *k-*mers is absent from the KrakenUniq report file when confidence scoring is applied and cannot be assessed.

In our assessment of Kraken 2 distinct *k*-minimizer metric, approximately 775 k*-*min were identified in the high spike-in RTE turkey deli meat dataset compared to over 2,300 k*-*min in the high spike-in prepackaged spinach dataset. This was consistent across reference databases. In the medium and low spike-in datasets, less than 160 k*-*min were detected across both sample types (Additional file 5). While the application of distinct *k*-minimizers for pathogen detection is promising, this feature is experimental and requires further validation with well-defined negative controls to determine the minimum number of distinct *k-*minimizers necessary to confidently support a positive pathogen detection result [49].

### Fewer computational resources are required for classification using a taxon-specific reference database

**Fig. 4:**
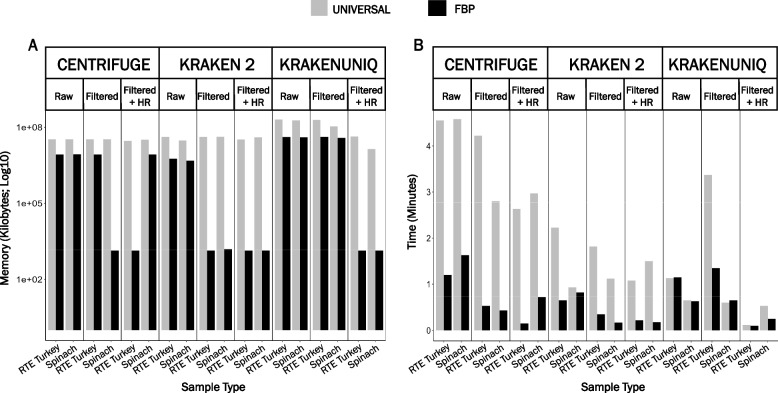
Comparison of computational resources. Resources were assessed based on [**A**] memory (kilobytes) and [**B**] time (minutes) required to process ready-to-eat (RTE) turkey deli meat and prepackaged spinach artificially contaminated with *L. monocytogenes* colony forming units (CFU) at a high spike-in level (10^4^ CFU/ml) using 8 cores. Raw = raw datasets classified with default settings (RTE turkey = 1,026,107 reads; Spinach = 417,409 reads); fastp = low-quality read filtered datasets classified with default settings (RTE turkey = 963,262 reads; Spinach = 363,404 reads); fastp + HR = low-quality read filtered (fastp) and in silico host removed (Bowtie 2) datasets classified with default settings (RTE turkey = 27,236 reads; Spinach = 341,848 reads). UNIVERSAL = universal (taxon-agnostic) reference database; FBP = foodborne pathogen-specific (taxon-specific) reference database. *KrakenUniq reference databases were preloaded prior to taxonomic classification using the *--preload* option

On average, memory usage and runtime were lower when using the FBP-specific database than the universal database, especially when using Kraken 2 for taxonomic classification (Fig. 4). Performing taxonomic classification on the same dataset immediately following the first run improved runtime across both reference databases for all classification software (data not shown). This finding was consistent with a previous study conducted by Ye et al*.* and is attributed to efficient database caching, which maps the database files into memory [25]. Adjusting the analysis parameters had a negligible impact on memory usage and runtime and, therefore, was not reported.

## Discussion

Reference databases perform well when taxa in a dataset are genetically distinct from one other and genetically similar to genomes in the reference database [50]. However, when there is insufficient diversity in the reference database, especially in the case of taxon-specific databases, it can lead to a considerable amount of non-specific read classification, which can be further exacerbated if even a small fraction of contamination is present in the reference genomes [19, 51]. This problem is particularly acute when using short reads. Several studies have emphasized the presence of source host DNA in genomes deposited in large-scale sequence repositories as contaminants, including RefSeq and GenBank, which are two key resources used for building reference databases [19–23]. If not dealt with appropriately, host DNA can become particularly challenging for accurate pathogen detection, especially when only a limited fraction of the DNA within a sample represents the pathogen of interest, as is often the case with contaminated foods. In this study, we show that in silico removal of host DNA, and to a lesser extent, low-quality read filtering was valuable for improving classification precision when conducting metagenomics-based pathogen detection on datasets with elevated host content using an FBP-specific reference, such as RTE turkey deli meat. Despite equivalent spike-in volumes across both sample types, we presume the high host DNA content coupled with a lower microbial load in the RTE turkey deli meat datasets led to fewer TP classifications across both reference databases compared to prepackaged spinach datasets.

Host DNA contamination is not unique to foods and is also problematic for pathogen detection in clinical specimens [52–54]. Fortunately, host-read removal in silico can improve pathogen detection, as demonstrated in this study. However, unwanted sequencing of host reads incurs costly and time-consuming computational host-read subtraction [55]. To overcome this challenge, several commercial-based and in-house wet-laboratory methods have been developed and evaluated for host DNA depletion in various clinical specimens, with some capable of removing up to 99.9% of host DNA [52–54]. Though promising, Ganda et al*.* demonstrated that these methods are not guaranteed to work with all sample types, particularly foods, and must be validated according to the pathogen and matrix [56].

Since naturally contaminated food datasets were unavailable for this study, metagenomic datasets from artificially contaminated food datasets were used. Unlike *in-silico*-generated datasets, these datasets often contain sequence contamination arising from laboratory reagents, sequencing kits, and cross-contamination between samples, in addition to lower read counts for true positives, which makes the task of separating true and false positives more challenging, and therefore, more akin to biological datasets. Despite our efforts, it is important to recognize the challenges in interpreting the results from modelled experiments as they may not represent "true" contamination dynamics—a study limitation. For example, the *L. monocytogenes* strain used in this study was not exposed to a sub-lethal treatment (i.e., drying, heating, chilling or freezing) to simulate the manufacturing process before artificial contamination in RTE turkey deli meat, which is likely to cause injury to the target pathogen (if present) and further impact detection. Since the intent of the study was to address the challenges associated with reference databases for metagenomics-based pathogen detection, wet-laboratory optimization was not performed. Additionally, the samples selected for this study were not subjected to a pre-enrichment step, a standard practice for conventional pathogen detection in foods. This may have further affected detection independent of the reference database used. Despite this limitation, we show that when the appropriate quality control measures, classification software and analysis parameters were applied, comparable classification performance was obtained across both reference databases.

In this study, metagenomics-based pathogen detection of *L. monocytogenes* was more reliable in foods containing higher pathogen levels approaching the lower limit of detection of traditional, non-enrichment culture-based methods (~ 10^4^ to 10^5^ CFU/ml) in food [57]. While a 48-h enrichment period is traditionally applied for detecting and isolating *L. monocytogenes* in foods, evidence suggests that direct sequencing of overnight primary enrichment cultures (quasimetagenomics) can generate sufficient genomic information for source tracking of *L. monocytogenes* [15, 16]. If properly optimized to balance the cost (time) with the benefit (sufficient genomic data), such an approach could speed up and simplify workflows for FBP surveillance and outbreak detection, especially with a taxon-specific reference database.

The diagnostic value and impending role of metagenomics as a surveillance and outbreak detection tool have led to a drastic increase in classification tools and, correspondingly, benchmarking studies [25, 58–62]. While valuable, it is important to acknowledge that most of these studies were conducted using existing pre-computed default reference databases. Until recently, they did not consider using reference databases with consistent references and taxonomy across classifiers [25]. Because of this, it is difficult to say whether differences in classification performance were attributed to the reference database or the classification algorithm. Therefore, to eliminate any confounding effects that may arise from differences in default database composition, using reference databases with consistent reference sequences and taxonomy across classifiers is essential when conducting a comparative analysis—this was a major strength of this study.

Another common trend of benchmarking studies is the tendency to treat bioinformatics software as "black box" devices, wherein users commonly and blindly apply default parameters. However, careful consideration of analysis parameters in the context of the research question should be taken into account, as default parameters do not always provide the most optimal result. By design, Centrifuge, in its default state, makes use of both large (≥ 31 bp) and small (22 bp) *k-*mers to achieve a more desirable trade-off between sensitivity and precision. Short *k*-mer matches can be problematic when the genomes in the dataset do not have a close genetic match in the reference database, as in the case of a taxon-specific reference database. This is because shorter *k*-mers are less likely to be unique to a specific taxon; thus, spurious hits to multiple genomes are more likely to ensue. When classified with Centrifuge, nearly all FP classifications identified in preprocessed datasets generated from artificially contaminated prepackaged spinach samples arose from short *k-*mer matches. This scenario also explains why highly abundant bacterial sequences in the prepackaged spinach datasets (i.e., *Pseudomonas* spp.) contributed to a substantial amount of FP classifications and could not be resolved even when the sequence label threshold was adjusted from the default of five down to one. Only when the minimum length of partial hits was adjusted from 22 to 31 bp was an equivalent precision to Kraken 2 and KrakenUniq achieved for the FBP-specific reference database. Likewise, applying a confidence score threshold of 0.1 to datasets classified with Kraken 2 or KrakenUniq was necessary to achieve high classification precision when using the FBP-specific reference database (≥ 0.98). Interestingly, when the aforementioned analysis parameters were applied to the raw datasets classified with the FBP-specific reference database, high classification precision (≥ 0.92) and recall (> 0.90) were still achieved across all classifiers, though slightly lower than preprocessed datasets.

This study's findings indicate that removing non-informative reads and adjusting analysis parameters prior to metagenomics-based pathogen detection analysis is beneficial when using a taxon-specific reference database, and will reduce FP classifications and improve the true pathogen signal. Since optimization of the confidence score threshold was not conducted in this study, robust parameter testing is necessary to determine the most suitable threshold when using a taxon-specific reference database.

Ultimately, the classification approach implemented by Kraken 2 and KrakenUniq helped drastically reduce the risk of FP classifications by assigning shared sequences to the LCA among the set of matching taxa. While the LCA approach can be extremely useful for resolving misclassifications, the approach also tends to spread the taxonomic level of the classifications from the more specific to the more general. Therefore, as public sequence repositories grow (i.e., RefSeq), it will be important to continuously re-evaluate how classification performance is impacted by changes in the distribution of reference genomes per taxon [63]. Although this study focused on three popular metagenomic classifiers that can generate custom reference databases, many classification tools offer similar features that were not considered but can be assessed using this study as the experimental model.

Speed and memory requirements are often critical factors in analyzing large-scale datasets, especially when conducting metagenomics-based pathogen detection with large reference databases, which can require a significant amount of computational resources (i.e., RAM) depending on the classification software [25]. A recent survey of laboratories in low and middle-income countries participating in PulseNet International, a global network comprising 88 countries to track foodborne diseases, found that only 28% of laboratories have access to local high-performance computing [64]. This limited access highly restricts the type of data analysis that can be conducted in those laboratories. We demonstrate that equivalent precision can be attained using a smaller reference database when the appropriate quality control measures, classification software, and analysis parameters are applied. Although the highest level of precision and recall for metagenomic-based detection of *L. monocytogenes* was achieved when using KrakenUniq across both reference databases, the most optimal trade-off between classification performance and computational efficiency was achieved when using Kraken 2 and the FBP-specific reference database. Overall, this approach is less computationally demanding and more attainable for the broader scientific and food safety communities. Unfortunately, the advantages of a lower memory footprint offered by the smaller Kraken 2 reference database come at the cost of slightly lower classification performance [38].

At a minimum, applying a confidence-scoring threshold can improve classification precision when using Kraken 2. However, in scenarios that require very high precision, for example, in an outbreak response, where even a few FP can be detrimental, KrakenUniq may be preferred over Kraken 2 [65]. As a result, recent improvements to the KrakenUniq software now enable "database chunking" [65]. By loading the reference database in chunks according to the available memory, this feature enables users to perform taxonomic classification using KrakenUniq on virtually any modern computer. However, the trade-off with "database chunking" is the much slower classification speeds, especially for large datasets [65].

## Conclusions

A taxon-specific reference database can be used to reliably conduct metagenomics-based detection of *L. monocytogenes* in RTE turkey deli meat and prepackaged spinach when the appropriate quality control measures, classification software, and analysis parameters are applied. Such an approach will allow users with limited computational resources to perform similar analyses, which may help accelerate the use of metagenomics-based pathogen detection for food safety, surveillance, and outbreak detection.

## Methods

### Reference sequence data

Complete genomes for bacteria (*n* = 17,215), archaea (*n* = 351), viruses (*n* = 9,507), and human (GRCh38.p13; GCF_ 000,001,405.39) were downloaded from the NCBI RefSeq Database Release 98 (January 2020) using the *krakenuniq-download* script [39, 66]. Low-complexity sequence masking of all complete genomes was performed using *DustMasker* with default parameters [67]. Contaminant sequence databases, UniVec and EMVEC, were also downloaded using the *krakenuniq-download* script [39].

### Tool selection

Three popular classifiers, Centrifuge [37], Kraken 2 [38], and KrakenUniq [39], were selected for the study based on availability, usability, and adoption. All tools are freely available, well documented, widely adopted by the scientific community, and actively maintained and updated by developers. Additionally, these classifiers support custom database construction.

### Database creation

All metagenomic classifiers selected for the study require a pre-computed reference database containing previously sequenced microbial genomes. We built two custom databases with consistent reference sequences and taxonomy across all classifiers to limit potential confounding effects due to differences in pre-computed (i.e., default) databases. All complete bacterial, archaeal, viral, and human genomes were downloaded from RefSeq, including the contaminant databases, and were used to build a universal (taxon-agnostic) reference database for each of the three metagenomic classifiers. A subset of complete genomes from the original RefSeq download, corresponding to twenty of the top thirty foodborne bacterial and viral pathogens in Canada, was used to create a foodborne pathogen (FBP)-specific database for each of the three metagenomic classifiers [68]. Additional enteric pathogens, including other *Listeria* and *Helicobacter* species, were also included in the FBP-specific reference database based on interest within PulseNet Canada, the National Molecular Subtyping Network for Foodborne Disease Surveillance. The human genome and contaminant databases were also included in the FBP-specific reference database to provide a lower rate of false-positive classifications as previously recommended [69]. All databases were built according to the developer's guidelines with default parameters. To assess reference genome quality and detect reference sequence contamination in public databases, the FBP-specific reference database, which contains the same subset of foodborne bacterial pathogens included in the universal reference database, was assessed for contamination/ completion using checkM (Additional file 6) and for cross-kingdom contamination using conterminator (Additional file 4) [44, 70]. All reference genomes included in the universal and FBP-specific reference databases are listed in Additional file 7. Database size, build time, and memory requirements are detailed in Additional file 8.

### Test datasets

Naturally contaminated datasets were unavailable, so artificially contaminated datasets generated from a previous pilot study involving the authors were used [unpublished data]. Briefly, two food commodities reported in foodborne disease outbreaks of *L. monocytogenes*, including ready-to-eat (RTE) turkey deli meat and prepackaged fresh spinach, were artificially contaminated with colony-forming units (CFU) at three spike-in levels categorized as high (10^4^ CFU/ml), medium (10^3^ CFU/ml) and low (10^2^ CFU/ml) with a strain of *L. monocytogenes* (HPB5415; GCF_000712385.1) from a 2008 Canadian listeriosis outbreak [71]. This strain was isolated from the implicated food product (i.e., RTE turkey deli meat) in the 2008 Canadian listeriosis outbreak, and therefore, was selected to more closely resemble the true outbreak scenario. To streamline data analysis, the same outbreak strain (HBP5415) was used to artificially contaminate spinach. Following overnight growth, a single colony was inoculated into 200 mL of Brain Heart Infusion (BHI) broth and grown overnight at 35 °C ± 2 °C to yield approximately 10^9^ CFU/mL. For each food product, 25 g of food was added to 225 mL of UVM1 (primary enrichment broth) and stomached for 2 min at 260 rpm. Aliquots of food homogenate were artificially contaminated with serial dilutions of HPB5415 (prepared in peptone water) to achieve three spike-in levels categorized as high (10^4^ CFU/mL), medium (10^3^ CFU/mL) and low (10^2^ CFU/mL), which are consistent with the infectious dose of *L. monocytogenes* reported in healthy persons and persons with weakened immune systems [72]. Both food commodities were confirmed via overnight growth on RAPID'*L.mono* (Bio-Rad, Hercules, California) to be negative for *L. monocytogenes*. Due to limited information in the literature to support a single best protocol for genomic DNA (gDNA) extraction, three commercial kits demonstrating good performance in two comprehensive benchmarking studies involving clinical specimens were selected and assessed: 1) QIAamp® Fast DNA Stool Mini Kit (Qiagen, Valencia, California), 2) DNeasy® PowerSoil Kit (Qiagen), and 3) Quick-DNA Fecal/Soil Microbe Miniprep Kit (Zymo Research, Irvine, CA) [62, 73]. gDNA extraction was carried out according to the manufacturer's instructions; however, an initial bead-beating step (PowerBead Tubes 2 ml, Glass 0.1 mm; Qiagen) was performed using the Vortex-Genie mixer at maximum speed for ten minutes before gDNA extraction using the QIAamp® Fast DNA Stool Mini Kit (Qiagen). Datasets were processed in duplicate for each extraction kit. gDNA concentration was quantitated using the Qubit Fluorometer (ThermoFisher, Waltham, MA) and diluted to 0.2 ng/µL. Libraries were prepped and assessed for quality according to the manufacturer's instructions for the Nextera XT DNA Preparation Library Kit (Illumina). Sequencing was performed on the Illumina MiSeq using the Illumina MiSeq Reagent Kit v3 (600-cycle).

### Data preprocessing

A list of the software used in the study is summarized in Additional file 9. The general quality of the raw fastq files was assessed using FastQC with default parameters [74]. Adaptor removal and read trimming were performed using fastp with a stringent qualified quality Phred value (-q) adjusted to 20 (default 15) [75]. Host sequences were filtered using Bowtie 2 with custom-built indexes, one for the spinach genome (GCF_002007265.1) and the other for the turkey genome (GCF_000146605.3) [76]. Two unplaced scaffolds previously identified as contaminated in the spinach genome (RefSeq identifiers NW_018932190.1 and NW_018932355.1) were masked before building the index [44]. Only primary alignments with both reads unmapped were included in the host sequence removed datasets using the SAM flags -f 12, -F 256 [77].

### Metagenomic classification and verification

To identify reads mapping to *L. monocytogenes*, all datasets were subjected to metagenomic classification using all three classifiers with default parameters. To assess whether modifications to analysis parameters could improve classification precision and recall for the reference databases, the sequence threshold label (*k*) was adjusted from five to one with and without adjusting the minimum length of partial hits *(--min-hitlen*) parameter from a default of 22 bp to 31 bp for datasets classified with Centrifuge. For datasets classified with Kraken 2 or KrakenUniq, a confidence score threshold (*--confidence*) of 0.1 was applied. All classification results were loaded into Pavian [78]. To account for the heterogeneity in assigned taxonomy ID within a species, all reads classified as *L. monocytogenes* (taxID 1639), including children taxon (e.g. *L. monocytogenes* strains), were extracted from the respective fastq files using the *krakenuniq-extract-reads* script with the -t option [18, 39]. Extracted reads were aligned to the reference genome (HPB5415) using Bowtie 2 to verify the number of true positive and false positive reads [76]. In this study, reads classified as *L. monocytogenes* and uniquely aligning to the reference genome with a mapping quality score of 42 using Bowtie 2 were categorized as TP, whereas reads that were classified as *L. monocytogenes* but did not uniquely align to the reference genome were defined as FP. Reads uniquely aligning to the *L. monocytogenes* reference genome in the raw datasets (ground truth) but not classified as *L. monocytogenes* were defined as FN. All values were averaged across replicates before calculating precision, recall and F1-scores.

### Visualizations

Figures were generated in R Studio [79] using the R programming language [80] and ggplot2 R package [81].

## Supplementary Information

**Additional file 1.**

**Additional file 2.**

**Additional file 3.**

**Additional file 4.**

**Additional file 5.**

**Additional file 6.**

**Additional file 7.**

**Additional file 8.**

**Additional file 9.**

## Data Availability

All datasets are available in NCBI under BioProject PRJNA891282. The test datasets selected for analysis in the current study correspond to BioSample accessions SAMN31353922 to SAMN31353933. The universal and FBP-specific reference databases are available from the corresponding author upon request.

## Abbreviations

BLAST: Basic Local Alignment Search Tool
gDNA: Genomic DNA
FBP: Foodborne pathogen
FN: False negative
FP: False positive
FPE: Food processing environment
LCA: Lowest common ancestor
NCBI: National Center for Biotechnology Information
PE: Paired-end
RefSeq: Reference Sequence Database
RTE: Ready-to-eat
TP: True positive
